# Defining quantification methods and optimizing protocols for microarray hybridization of circulating microRNAs

**DOI:** 10.1038/s41598-017-08134-3

**Published:** 2017-08-10

**Authors:** Anna Garcia-Elias, Leonor Alloza, Eulàlia Puigdecanet, Lara Nonell, Marta Tajes, Joao Curado, Cristina Enjuanes, Oscar Díaz, Jordi Bruguera, Julio Martí-Almor, Josep Comín-Colet, Begoña Benito

**Affiliations:** 1 Group of Biomedical Research in Heart Diseases, IMIM (Hospital del Mar Medical Research Institute), C/Doctor Aiguader 88, 08003 Barcelona, Spain; 2 Microarray Analysis Service, IMIM (Hospital del Mar Medical Research Institute), C/Doctor Aiguader 88, 08003 Barcelona, Spain; 3 Cardiology Department, Hospital del Mar, Passeig Marítim 25–29, 08003 Barcelona, Spain; 4 Centre for Genomic Regulation (CRG), The Barcelona Institute of Science and Technology, Dr. Aiguader, 88, 08003 Barcelona, Spain; 5 Universitat Pompeu Fabra, C/Dr. Aiguader 88, 08003 Barcelona, Spain; 6 Cardiovascular Risk and Nutrition Group, IMIM (Hospital del Mar Medical Research Institute), C/Doctor Aiguader 88, 08003 Barcelona, Spain

## Abstract

MicroRNAs (miRNAs) have emerged as promising biomarkers of disease. Their potential use in clinical practice requires standardized protocols with very low miRNA concentrations, particularly in plasma samples. Here we tested the most appropriate method for miRNA quantification and validated the performance of a hybridization platform using lower amounts of starting RNA. miRNAs isolated from human plasma and from a reference sample were quantified using four platforms and profiled with hybridization arrays and RNA sequencing (RNA-seq). Our results indicate that the Infinite^®^ 200 PRO Nanoquant and Nanodrop 2000 spectrophotometers magnified the miRNA concentration by detecting contaminants, proteins, and other forms of RNA. The Agilent 2100 Bioanalyzer PicoChip and SmallChip gave valuable information on RNA profile but were not a reliable quantification method for plasma samples. The Qubit^®^ 2.0 Fluorometer provided the most accurate quantification of miRNA content, although RNA-seq confirmed that only ~58% of small RNAs in plasma are true miRNAs. On the other hand, reducing the starting RNA to 70% of the recommended amount for miRNA profiling with arrays yielded results comparable to those obtained with the full amount, whereas a 50% reduction did not. These findings provide important clues for miRNA determination in human plasma samples.

## Introduction

MicroRNAs (miRNAs) are small (18–25 nucleotides), non-coding RNA molecules that control gene expression by generally suppressing target messenger RNAs^1, 2^. They are thought to be involved in the regulation of nearly all cell functions. More than one third of the human transcriptome has been found to be targeted by at least one of the human miRNAs described so far (⋍2,600, increasing in number and annotated in the miRNA database, www.mirbase.org)^3, 4^. As crucial regulators of gene expression, miRNAs are involved in multiple disease processes^5, 6^. Although mostly expressed intracellularly, circulating miRNAs can be detected in blood and other organic fluids^7–10^, where they present a remarkable fluid stability^11–13^. Circulating miRNAs are thought to participate in cell-to-cell communication and distant regulation of gene expression, and, as such, have recently arisen as promising biomarkers for early detection of disease, prognostic stratification, and identification of potential therapeutic targets^12, 14–17^. Although an exciting prospect, the isolation and profiling of miRNAs from body fluids remains challenging, mostly due to their extremely low concentration^14^, which can be particularly relevant when using microarray technology. Differences in sample extraction, miRNA isolation, quantification, or profiling methods may yield inexact and/or non-reproducible results^6, 18, 19^. Therefore, accurate protocols must be defined before circulating miRNAs can be definitely established as clinical biomarkers^4, 7, 20, 21^. In order to normalize methods, efforts have been made to compare different miRNA isolation^5, 22, 23^ or profiling platforms^15^ but, to our knowledge, no study has aimed to standardize miRNA quantification in plasma. Previous work by Deben *et al*. comparing different quantification methods suggested that fluorometric analysis could be more suitable than spectrophotometry for RNA quantification in tissue samples^24^. However, whether this applies to miRNA quantification and extends to plasma samples, where RNA content is approximately 10 times lower^25^, remains to be established.

Considering that some profiling platforms require a specific amount of starting material, optimal miRNA quantification in plasma samples is a critical step to obtain valid results. On the other hand, this initial amount of RNA may be difficult to achieve, due to the low amount of these molecules in peripheral blood, which is important when using certain platforms such as array hybridization assays. Our work addressed both limitations.

We first sought to define the most appropriate method for miRNA quantification in plasma samples, using the Universal Human miRNA Reference (miRNA-Ref) from Agilent Technologies as a reference sample. After comparing four widely used quantification platforms (Tecan Infinite^®^ 200 PRO Nanoquant Spectrophotometer, Thermo Scientific Nanodrop 2000 Spectrophotometer, Agilent 2100 Bioanalyzer, and Life Technologies Qubit^®^ 2.0 Fluorometer), we found that both spectrophotometers overestimated the miRNA content in both types of samples, compared to the fluorometric technique, by detecting other nucleotides, proteins, and contaminants that could have been isolation-derived. This was confirmed by the results of the smear analysis of the electropherograms obtained with the Agilent 2100 Bioanalyzer, although this technique was discarded as a valid quantification platform for plasma because of its high variability. Therefore, in our hands, Qubit^®^ 2.0 Fluorometer was the most suitable technique for specific miRNA quantification, due to its higher specificity for small RNA molecules. Nonetheless, RNA-seq of our plasma samples showed that, among the small RNAs, only 58% of the total counts are true miRNAs, highlighting the limitations of all current quantification techniques to estimate the true miRNAs content.

To overcome the potential limitation of not having enough starting RNA when using microarrays with low miRNA-content samples, we tested the performance of GeneChip^®^ miRNA 3.0 and 4.0 platforms (Affymetrix) using lesser starting amounts of material than those specified by the manufacturer protocol. We confirmed that using 70% of the recommended starting RNA (but not a 50% reduction) had overall good performance for plasma samples, comparable to that obtained when following the original protocol.

## Results

### RNA profile and integrity

**Figure 1:**
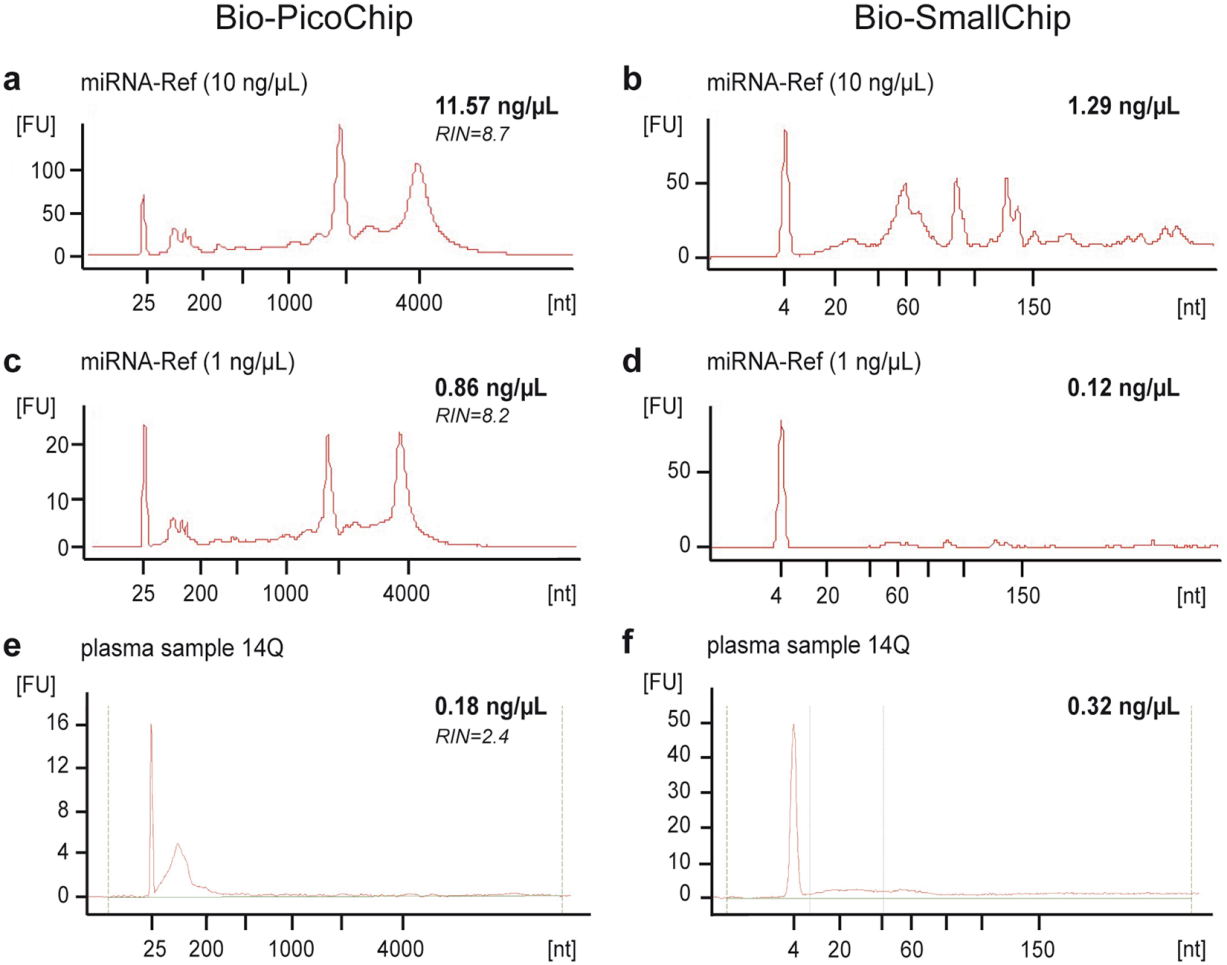
Electropherograms obtained with the Bioanalyzer 2100 in miRNA-Ref and plasma samples. Examples of the profiles obtained from a miRNA-Ref sample at 10 ng/μL (**a**, Bio-PicoChip; (**b**), Bio-SmallChip), a miRNA-Ref sample at 1 ng/μL (**c**, Bio-PicoChip; (**d**), Bio-SmallChip), and a plasma sample (**e**, Bio-PicoChip; (**f**), Bio-SmallChip). The 10 ng/µL miRNA-Ref sample (**a**) was diluted to fit the detection range of the Bio-PicoChip (0.05–5 ng/µL) and the final concentration was calculated by applying the dilution factor to the value obtained by the Bioanalyzer. In the miRNA-Ref samples (**a**–**d**), the overall profile is consistent with the presence of ribosomic RNA enriched with small RNAs, whereas in plasma samples (**e**,**f**) only small RNAs, but no long RNAs, are present. All electropherograms include the corresponding quantification (in bold) and the RNA integrity number (RIN) number (in italics) obtained with the Bio-PicoChip (**a**,**c**,**e**) or just the quantification (in bold) with the Bio-SmallChip (**b**,**d**,**f**).

Examples of the different sample profiles analysed with the Agilent 2100 Bioanalyzer RNA 6000 PicoChip (Bio-PicoChip) and SmallChip (Bio-SmallChip) are illustrated in Fig. 1. Visual inspection of Bio-PicoChip results confirmed the presence of ribosomic RNA in the miRNA-Ref samples at both concentrations tested (10 ng/µL and 1 ng/μL), consistent with a total RNA profile enriched with miRNAs. As expected, only small RNAs (<200 nt) and no long RNAs were present in plasma samples subjected to miRNA isolation (miRNeasy, Qiagen). RNA integrity number (RIN) values were much higher in miRNA-Ref samples than in plasma samples, consistent with the absence of 18 S and 28 S ribosomic RNA species in the latter.

### Quantification

#### miRNA Reference RNA

Quantification results of the two miRNA-Ref working dilutions (10 ng/µL and 1 ng/µL) by the Nanoquant, Nanodrop, Qubit, Bio-PicoChip, and Bio-SmallChip platforms are presented in Table 1. First, we checked the repeatability of the results by estimating the coefficient of variation (CV) of 10 consecutive measurements of identical samples at both concentrations on all platforms. As seen in Table 1, at 10 ng/µL, repeatability was acceptable with the first four platforms (CVs of 2.03–5.78%) but more variable with the Bio-SmallChip (CV of 12.25%). At 1 ng/µL, results with the Nanoquant and the Nanodrop were notably variable (CVs of 40.0% and 23.8%, respectively), but remained highly reproducible with the Qubit platform (CV 2.81%). The explanation for this discrepancy is that 1 ng/µL is below the detection threshold of both the Nanoquant (3 ng/µL) and Nanodrop (2 ng/µL) assays, but remains within the Qubit range (0.05–100 ng/µL). With the Bio-PicoChip and Bio-SmallChip, variability at 1 ng/μL was also high (CV of 8.26% and 44.11%, respectively) despite being within the qualitative detection range of both chips, indicating a potential limitation of this technique when quantifying low-concentration samples.

**Table 1:** RNA contents in miRNA-Ref samples and repeatability of four quantification platforms.

Working dilutions		Quantification platforms (ng/µL)				
		Nanoquant	Nanodrop	Qubit	Bio-PicoChip	Bio-SmallChip
10 ng/µL	1	8.64	10.30	2.40	11.57	1.07
	2	8.96	11.30	2.28	13.49	1.15
	3	10.16	11.00	2.42	12.25	1.14
	4	9.20	11.20	2.32	13.61	1.07
	5	9.28	10.90	2.36	12.75	1.12
	6	9.20	11.00	2.32	12.56	1.18
	7	8.64	10.70	2.28	13.47	1.29
	8	8.24	10.60	2.30	13.07	1.42
	9	8.72	10.90	2.34	13.15	1.35
	10	8.88	11.00	2.38	13.25	1.38
*Mean* (*SD*)		***8***.***99*** (***0***.***52***)	***10***.***93*** (***0***.***22***)	***2***.***34*** (***0***.***05***)	***12***.***92*** (***0***.***64***)	***1***.***22*** (***0***.***15***)
*CV* (%)		***5***.***78***	***2***.***03***	***2***.***09***	***4***.***98***	***12***.***25***
1 ng/µL	1	0.64	1.60	0.19	0.83	0.07
	2	1.04	0.70	0.19	0.94	0.06
	3	0.48	0.90	0.19	0.86	0.14
	4	0.96	1.10	0.18	0.81	0.07
	5	0.56	0.80	0.18	0.79	0.06
	6	0.80	1.20	0.18	0.78	0.06
	7	0.48	1.10	0.18	0.78	0.09
	8	0.24	1.10	0.18	0.70	0.12
	9	0.40	1.30	0.19	0.74	0.18
	10	0.64	1.00	0.18	0.77	0.15
*Mean* (*SD*)		***0***.***62*** (***0***.***25***)	***1***.***08*** (***0***.***26***)	***0***.***18*** (***0***.***01***)	***0***.***80*** (***0***.***07***)	***0***.***10*** (***0***.***04***)
*CV* (%)		***40***.***00***	***23***.***83***	***2***.***81***	***8***.***26***	***44***.***11***

**Figure 2:**
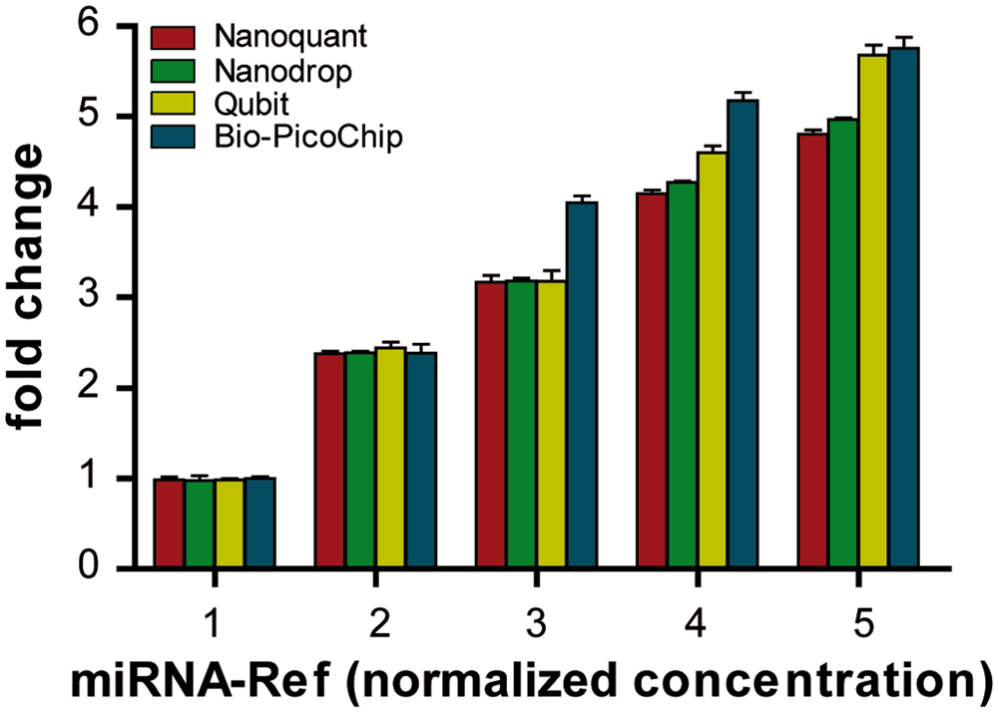
Quantification of miRNA-Ref samples at different normalized concentrations assessed by Nanoquant, Nanodrop, Qubit and Bio-PicoChip. Evaluation of the performance of the four quantification techniques in a series of five increasing miRNA-Ref concentrations prepared from the 10 ng/μL working solution. Data on the X axis are normalized to the lowest concentration (data in Supplementary Table S1). Values for all four platforms increased proportionally with increasing concentrations.

Notably, quantification results with the Nanoquant, Nanodrop, and Bio-PicoChip platforms were closer to those expected for each of the working dilutions (Table 1, mean values of 8.99 ng/µL, 10.93 ng/µL and 12.92 ng/µL for the 10 ng/µL dilution, and 0.62 ng/µL, 1.08 and 0.80 ng/µL at 1 ng/µL, respectively). Quantification with the Qubit platform yielded lower values, compared to the other three techniques, and at a similar proportion for all RNA concentrations. Nanoquant, Nanodrop, and Bio-PicoChip were 3.4–3.8, 4.7–5.9, and 4.4–5.5 times higher, respectively). Because the miRNA-Ref is composed of total RNA enriched with miRNAs, the quantifications provided by spectrophotometer-based techniques such as Nanoquant and Nanodrop most likely include RNA species other than miRNA (all being detected by absorbance at 260 nm). Accordingly, these two platforms provided values similar to the results from Bio-PicoChip, the electropherogram of which includes all RNA molecules ranging from 0 to 4000 nucleotides (Fig. 1a and c). Unlike these techniques, Qubit uses specific fluorescent dyes selective for small RNA over other forms of RNA, and therefore the concentration values obtained with this platform should correspond mainly to the portion of small RNAs present in the miRNA-Ref. Accordingly, the analysis of the Bio-SmallChip electropherograms, which only include molecules with less than 200 nt (Fig. 1b and d), yielded concentration results that were closer to the Qubit both at the 10 ng/µL and at the 1 ng/μL working dilutions (Table 1). In both cases, the concentration values estimated by the Bio-SmallChip were slightly lower than the Qubit values. This could be explained by a slight Qubit overestimation; this technique, which is highly sensitive for small RNA species, may still detect a small portion of molecules up to 1000 nt-long. However, it is important to note that Bio-SmallChip data were remarkably more variable than those of Qubit (Table 1, CVs of 12.25% and 44.11% versus 2.09% and 2.81% for the 10 ng/µL and the 1 ng/μL concentrations, respectively), limiting the use of the Bio-SmallChip as a reliable method for miRNA quantification. Importantly, all quantification platforms (except for the Bio-SmallChip that was not tested due to its high variability) maintained a notably stable relationship between their results at different concentrations of miRNA-Ref (Fig. 2, data in Supplementary Table S1).

#### Plasma samples

Plasma samples from healthy controls (1Q–20Q) were analysed with the same methodologies used for the miRNA-Ref (Fig. 1e and f, and Table 2). The overall variability in RNA content of our population was measured with the CV. Nanoquant, Nanodrop, and Qubit provided similar CV values (29.31%, 26.22%, and 26.16%, respectively). Nevertheless, Bio-PicoChip and especially Bio-SmallChip yielded much higher CVs (67.16% and 82.63%, respectively).

**Table 2:** RNA contents in plasma samples according to four quantification platforms.

ID plasma pools	Quantification platforms (ng/µL)					RIN
	Nanoquant	Nanodrop	Qubit	Bio-PicoChip	Bio-SmallChip	
1Q	7.04	9.40	1.60	0.423	0.372	1.7
2Q	6.64	9.57	1.46	0.142	0.281	1.5
3Q	14.48	18.70	0.90	0.091	0.126	2.1
4Q	14.64	18.90	0.98	0.152	0.149	2.1
5Q	8.32	11.23	1.17	0.250	0.120	3.2
6Q	6.40	8.10	0.92	0.088	0.065	1.1
7Q	7.28	9.30	0.81	0.131	0.009	1.1
8Q	6.16	10.10	0.76	0.082	0.031	1.2
9Q	5.84	8.50	0.81	0.091	0.059	1.5
10Q	9.76	11.90	0.72	0.069	0.017	1.3
11Q	5.84	7.10	0.76	0.079	0.072	1.4
12Q	10.08	13.50	1.65	0.103	0.180	1.3
13Q	8.16	11.10	0.82	0.090	—	1.4
14Q	8.64	10.90	1.31	0.175	0.324	2.4
15Q	9.84	11.60	1.04	0.091	0.251	1.3
16Q	11.04	13.80	1.12	0.250	0.210	NA
17Q	6.64	13.30	1.14	0.352	—	2.5
18Q	8.24	13.10	1.04	0.082	0.000	1.4
19Q	10.08	13.90	1.35	0.092	0.049	1.8
20Q	10.64	13.50	1.10	0.094	0.139	1.5
*Mean* (*SD*)	***8***.***79*** (***2***.***58***)	***11***.***87*** (***3***.***11***)	***1***.***07*** (***0***.***28***)	***0***.***146*** (***0***.***098***)	***0***.***136*** (***0***.***112***)	—
*CV* (%)	***29***.***31***	***26***.***22***	***26***.***16***	***67***.***16***	***82***.***63***	—

RIN: RNA integrity number.

As seen with the miRNA-Ref samples, Nanoquant and Nanodrop provided higher RNA concentration values compared to those obtained with the Qubit technique. In this case, the difference between techniques was greater (8- to 11-fold difference for Nanoquant or Nanodrop *vs*. Qubit), compared to that obtained for the miRNA-Ref (3- to 6-fold difference for Nanoquant or Nanodrop *vs*. Qubit). Assuming a more specific detection of miRNAs by the Qubit, compared to the other techniques, these results would be consistent with the type of samples used in each case: the miRNA-Ref is a manufactured product specifically enriched in miRNAs with low levels of isolation-derived contaminants – or none; plasma samples can be expected to have lower concentrations of miRNAs among other RNA species, degraded nucleotide molecules, proteins, and contaminants derived from the isolation process that could be detected by the spectrophotometer. To assess this possibility, we evaluated the 260/230 and 260/280 ratios provided by the Nanodrop as an indirect measure of RNA purity, where values ≥2 indicate pure RNA. Plasma samples had a mean 260/230 ratio of 0.14 ± 0.02 and a mean 260/280 ratio of 1.22 ± 0.03 compared to the 1.99 ± 0.12 and 1.98 ± 0.11 obtained with the miRNA-Ref samples (Supplementary Table S2). This indicates the presence of contaminants eluted with the miRNAs in plasma samples.

**Figure 3:**
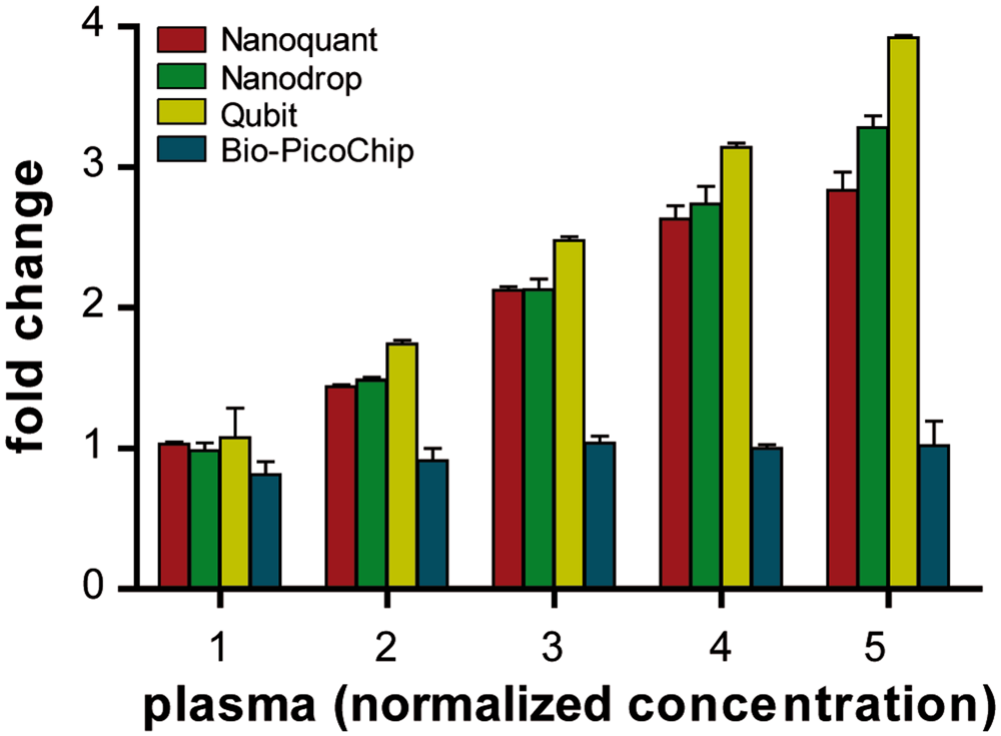
Quantification of plasma samples at different normalized concentrations assessed by Nanoquant, Nanodrop, Qubit and Bio-PicoChip. Evaluation of the performance of the four quantification techniques in a series of five increasing concentrations prepared from the pooled plasma. Data on the X axis are normalized to the lowest concentration (data in Supplementary Table S3). Bio-PicoChip results showed high variability and did not increase proportionally with increasing concentrations.

Bio-PicoChip and Bio-SmallChip provided notably lower values than those obtained with the Qubit (Table 2). Again, this difference could be explained by partial overestimation by the Qubit (which might include other forms of small RNAs besides miRNAs) and/or by the Bio-techniques. However, it is again important to highlight the high variability observed in the electropherogram analyses, particularly for the Bio-SmallChip, which would argue against the use of these methods for reliable miRNA quantification in plasma samples. Moreover, unlike the other techniques, the Bio-PicoChip failed to increase proportionally when different plasma concentrations were prepared (Fig. 3 and Supplementary Table S3).

**Figure 4:**
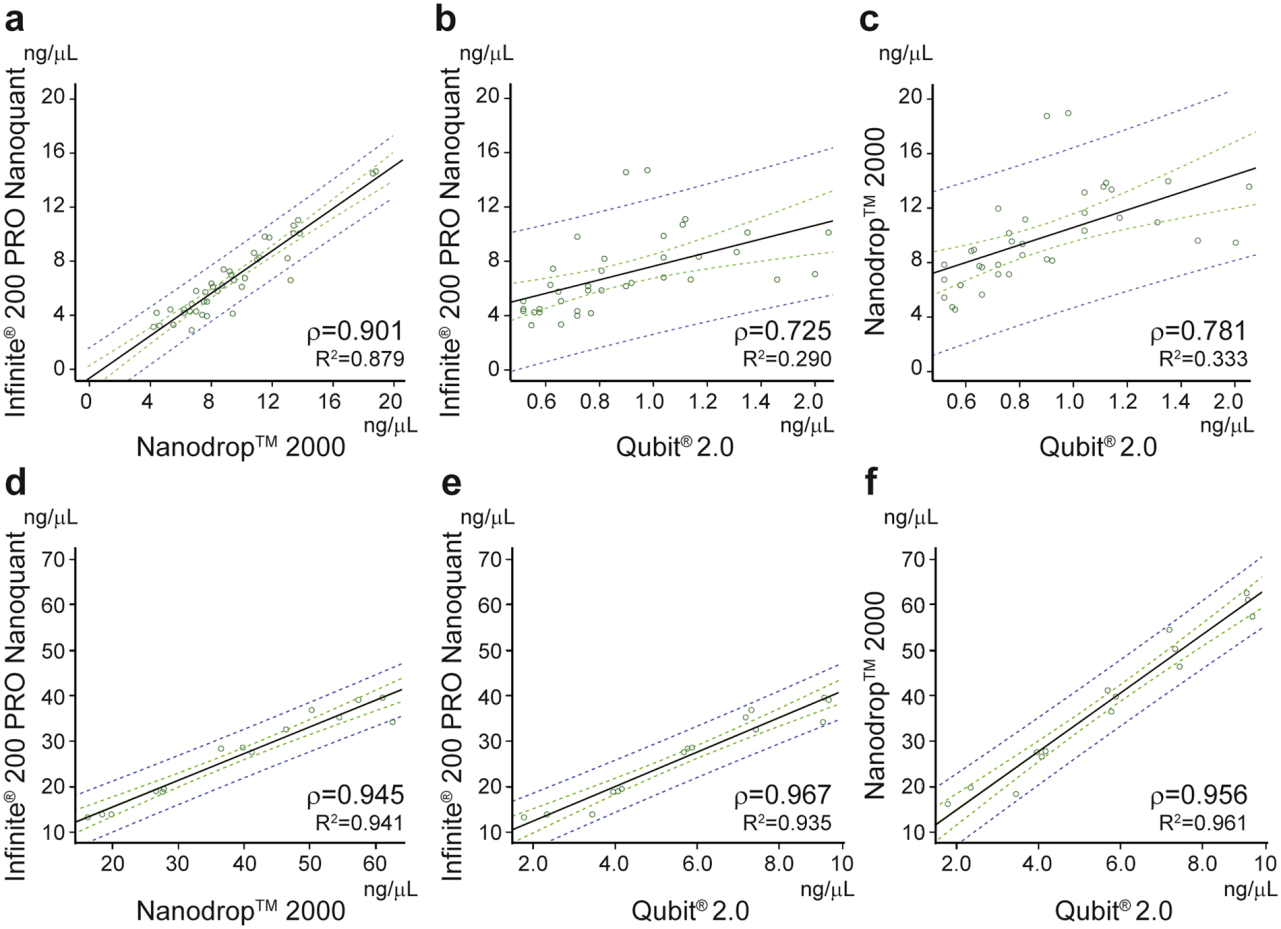
Correlations between Nanoquant, Nanodrop and Qubit. Correlations between techniques using original and diluted plasma samples (**a**–**c**). Correlations between techniques using concentrated plasma samples ≥2 ng/µL (**d**–**f**). Regression lines (black) with their 95% confidence interval (dashed green) and 95% prediction interval (dashed blue) were generated from the correlation of n = 41 (**a**–**c**) and n = 15 (**d**–**f**) quantification values. Correlations were assessed with Spearman rank correlation coefficient (ρ) and linear regression R^2^. Concentrations are expressed in ng/µL.

From these data, it appears that the Qubit platform might be the most appropriate methodology for miRNA quantification in plasma samples, given the potential detection of contaminants and RNA species other than miRNA with absorbance-based techniques and the variability observed with the smear analysis of Bio-techniques. However, because both the Nanoquant and (especially) the Nanodrop spectrophotometers are used in most laboratories, we checked for a good correlation between these techniques and the Qubit. To increase the range of concentrations, we prepared several solutions by diluting (by 1/2 and by 1/3) and concentrating (x2, x4, x6, x8 and x10) a subset of plasma samples from the pool of patients. Correlation results are depicted in Fig. 4. At the original plasma concentration or in diluted samples, although Nanoquant and Nanodrop kept good correlation with one another (ρ = 0.901 and R^2^ = 0.879) (Fig. 4a), both displayed only a modest correlation with Qubit: ρ = 0.725 and R^2^ = 0.290 for Qubit versus Nanoquant (Fig. 4b) and ρ = 0.781 and R^2^ = 0.333 for Qubit versus Nanodrop (Fig. 4c). Nevertheless, in more concentrated samples (>2 ng/μL by Qubit) correlation between the three techniques was very good: ρ = 0.945 and R^2^ = 0.941 for Nanoquant versus Nanodrop (Fig. 4d), ρ = 0.967 and R^2^ = 0.935 for Qubit versus Nanoquant (Fig. 4e) and ρ = 0.956 and R^2^ = 0.961 for Qubit versus Nanodrop (Fig. 4f). Again, this could be explained because plasma samples at their original concentration (≤1 ng/μL by Qubit) are below the detection range of the Nanodrop and Nanoquant, where neither platform seems to provide reliable results.

### Small RNA composition

One limitation of the Qubit methodology is its potential to detect all forms of small RNAs. Therefore, we decided to further characterize the small RNA content and particularly the miRNA content of both miRNA-Ref and plasma samples. We first estimated the proportion of short RNAs (20–40 nucleotides long, and thus, potential candidates to be miRNAs) among all small RNAs by analysing the Bio-SmallChip electropherograms. For the 10 ng/µL miRNA-Ref working dilution, the area under the curve (AUC) of the 20–40-nt region represented an average of 22% (±1.58%) of all small RNAs (Supplementary Table S4). For plasma samples, the AUC of the 20–40 nucleotides region was almost equivalent to the AUC of the total small RNA, given that all these samples had been subjected to miRNA extraction, although Bio-SmallChip results were more variable in this case.

We then generated a library of all small RNAs, and ran both the miRNA-Ref and plasma samples on an electrophoresis gel to characterize the different RNA species. Consistent with the Bio-SmallChip results, plasma samples displayed a single band at the 20–40 nt region, whereas the miRNA-Ref documented different small RNA species. To specifically characterize the miRNA content, we performed a RNA–seq of the fragment that includes all molecules between 20 and 40 nucleotides, and therefore mainly miRNAs. Reads with more than 2 multimaps were not considered. In total, 53% of the reads of the plasma sample and 39% of the miRNA-Ref sample were not mapped and therefore discarded. Table 3 shows the distribution of the read counts of different RNA species in both samples. In the miRNA-Ref sample, the proportion of miRNAs detected by RNA-seq in the 20–40 nucleotide region was 84.4%. In plasma, miRNAs represented 58.2% of all counts, followed by miscellaneous RNAs (misc_RNA: 12.8%). Of note, up to 28% of the counts were mapped in regions not previously annotated. Considering that the 20–40 nucleotide region represented the total small RNA population in plasma samples, the 58% result likely represents the actual proportion of miRNAs among small RNAs. These results confirm that RNA species other than miRNA are present in biological samples, even when restricting the analysis to very specific regions determined by nucleotide size, and highlight the potential limitations to the accuracy of all techniques, including Qubit, for miRNA quantification.

**Table 3:** Small RNA spectrum found in plasma and miRNA-Ref samples.

Type	miRNA-Ref		plasma	
	counts	%	counts	%
discarded reads (*)		39%		53%
scaRNA	299	0.1%	217	0.1%
ribozyme	27	0.0%	0	0.0%
misc_RNA	3992	1.0%	33695	12.8%
rRNA	634	0.2%	384	0.1%
vaultRNA	6	0.0%	0	0.0%
sRNA	11	0.0%	0	0.0%
miRNA	347441	84.4%	153596	58.2%
snoRNA	9431	2.3%	197	0.1%
tRNA	12660	3.1%	1663	0.6%
sRNA	536	0.1%	323	0.1%
unannotated	36794	8.9%	73817	28.0%
total counts (**)	411831	100%	263892	100%

(*) Unmapped reads and more than 2 multimaps of all sequenced reads. (**) Counts includes only mapped reads with 2 or less multimaps. scaRNA: small-Cajal body-specific RNA; misc_RNA: miscellaneous RNA; rRNA: ribosomic RNA; sRNA: 5 S ribosomal RNA; snoRNA: small nucleolar RNA; tRNA: transfer RNA; snRNA: small nuclear RNA.

### miRNA expression profiles

Expression profiles were checked with the GeneChip arrays from Affymetrix, which require an initial 130 ng of RNA. Of note, for those samples with a starting plasma volume of 2 mL (n = 11), the final RNA amounts obtained and quantified with the Qubit assay ranged from 95.94 ng to 192.58 ng, with a mean of 133.31 ± 26.50 ng (Table 4). Only 6 out of 11 samples (54.5%) reached the pre-specified minimum value of 130 ng. We thus performed the arrays with the starting amounts of 92 ng (70%) and 65 ng (50%), given that these could be achieved by all samples when miRNA isolation began with 2 mL plasma, a standard volume commonly used for storage in most clinical studies.

**Table 4:** RNA total amounts obtained from plasma samples according to the Qubit assay.

ID plasma pool	Starting plasma volume (µL)	Sample volume after RNA isolation (µL)	RNA quantification by Qubit (ng/µL)	Total RNA content (ng)
1Q	400	28	1.60	44.74
2Q	400	28	1.46	40.88
3Q	1200	72	0.90	64.80
4Q	1600	100	0.98	98.00
5Q	1200	72	1.17	84.24
6Q*	2000	132	0.92	121.44
7Q*	2000	132	0.81	107.45
8Q	1600	104	0.76	79.25
9Q	1600	104	0.81	84.66
10Q	1200	76	0.72	55.02
11Q	1200	76	0.76	57.91
12Q*	2000	117	1.65	192.58
13Q*	2000	117	0.82	95.94
14Q*	2000	117	1.31	152.88
15Q*	2000	117	1.04	121.45
16Q*	2000	117	1.12	131.35
17Q*	2000	117	1.14	133.61
18Q*	2000	117	1.04	121.99
19Q*	2000	117	1.35	158.42
20Q*	2000	117	1.10	129.25
*Mean* (*SD*)^*#*^			***1***.***12*** (***0***.***25***)	***133***.***31*** (***26***.***50***)

^#^Mean and SD values of samples with starting plasma volume of 2 mL (denoted with*).

**Figure 5:**
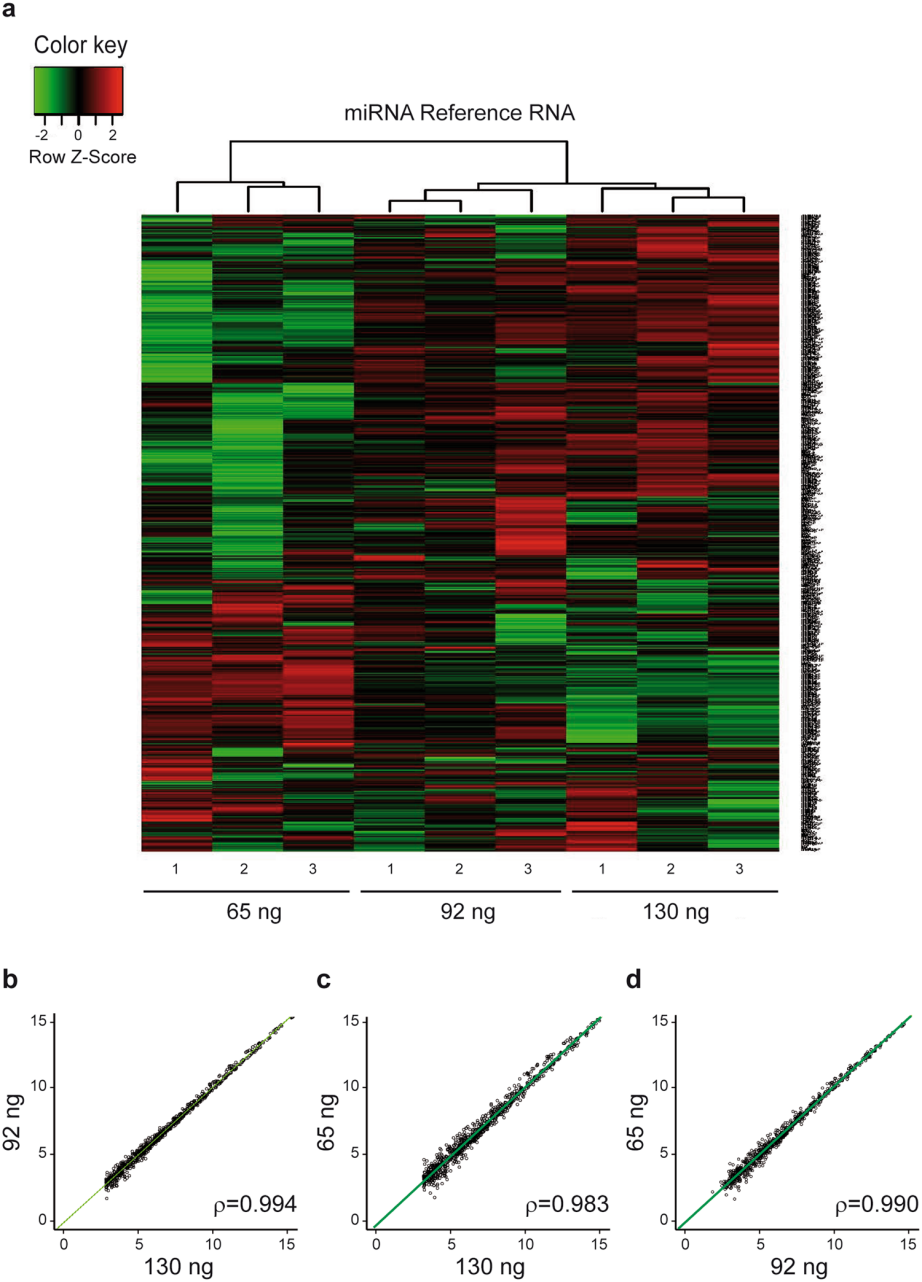
miRNA Reference RNA hybridization microarrays. (**a**) Heat maps obtained from three 130/, 92/and 65/miRNA-Ref hybridization microarrays. Only miRNA with significant expression (>3) in the 130 ng sample and observed in all three arrays are represented. (**b**) Correlation between the miRNA profiles of the mean of the triplicates for the three starting amounts. The regression line is represented as a solid green line. Axis units are expressed in log2 intensities.

We first performed three replicates of hybridization microarrays of the miRNA-Ref using the three different starting RNA amounts (Fig. 5, 130/miRNA-Ref, 92/miRNA-Ref, and 65/miRNA-Ref). According to our gold standard (130/miRNA-Ref), 615 miRNAs were considered to be expressed (Supplementary Table S5). Visual inspection of heat map results filtered for expressed miRNA (Fig. 5a) suggests a closer relationship between 130/miRNA-Ref and 92/miRNA-Ref arrays versus the 65/miRNA-Ref array, as depicted by the upper dendrogram. Analysis of correlation at the three starting material quantities tested was also consistent with this hypothesis, with ρ values of 0.994 for correlation between 130/miRNA-Ref and 92/miRNA-Ref (Fig. 5b), 0.983 for correlation between 130/miRNA-Ref and 65/miRNA-Ref (Fig. 5c), and 0.990 for correlation between 92/miRNA-Ref and 65/miRNA-Ref (Fig. 5d). Considering our gold standard (130/miRNA-Ref), we measured the sensitivity and specificity of the hybridization arrays performed with the other amounts. The results showed a 0.978 sensitivity and 0.997 specificity for the 92/miRNA-Ref array, whereas the respective values for the 65/miRNA-Ref array were 0.948 and 0.996.

**Figure 6:**
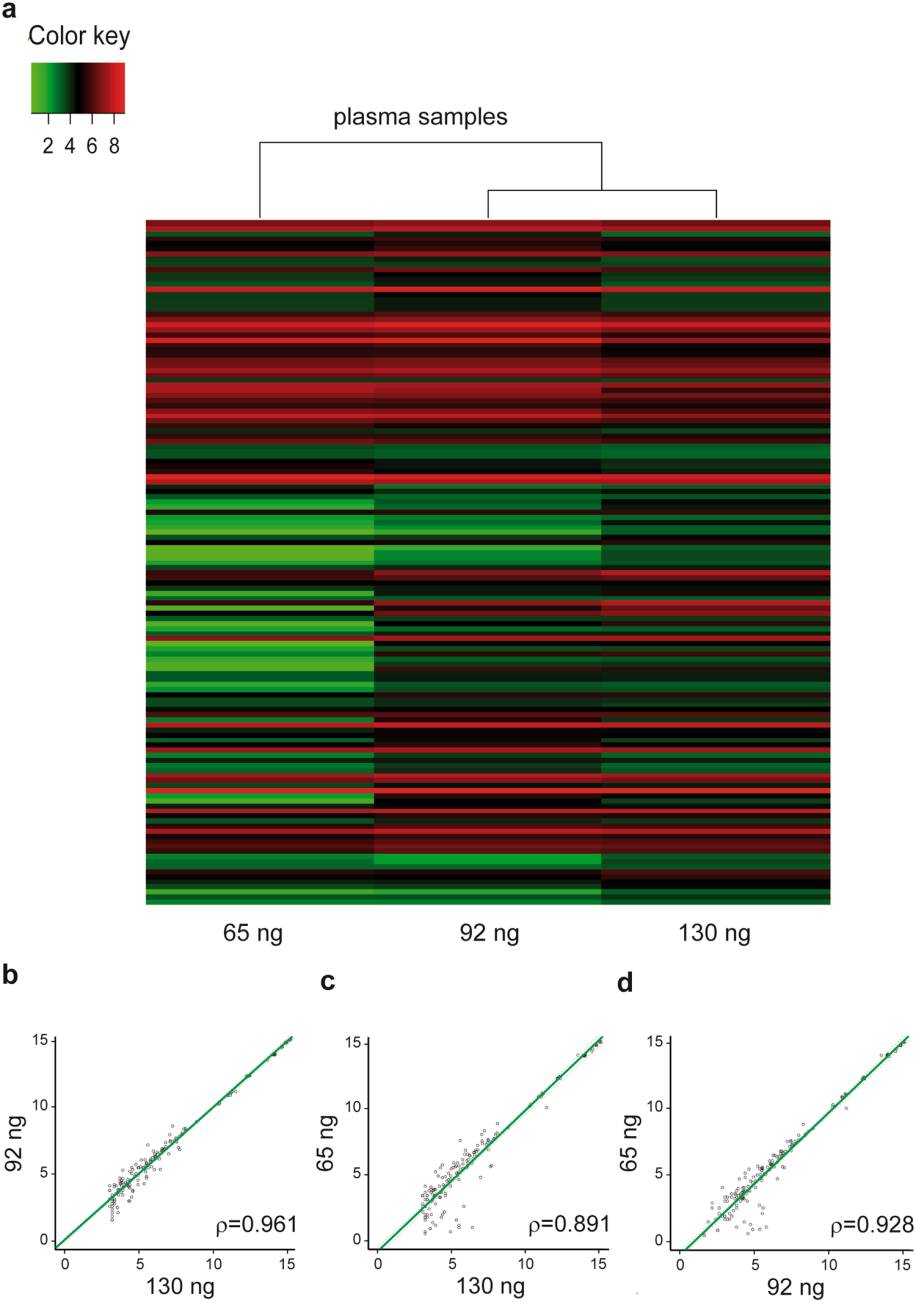
Plasma samples hybridization microarrays. (**a**) Heat maps obtained from the 130/, 92/, and 65/plasma hybridization microarrays. Only miRNA with significant expression (>3) in the 130 ng sample are represented. (**b**) Correlation between the miRNA profiles of three starting amounts. The regression line is represented as a solid green line. Axis units are expressed in log2 intensities.

To test whether these results could be extended to plasma samples, we performed three hybridization microarrays with the plasma samples obtained from healthy controls, using the same three starting RNA amounts (130/plasma, 92/plasma, and 65/plasma). Heat map and correlation plots filtered for expressed miRNAs are shown in Fig. 6. An important initial finding is that the amount of miRNAs expressed in plasma samples was notably inferior to that obtained with miRNA-Ref (118 versus 615, Supplementary Table S6). This is consistent with the different nature of both samples, with one being enriched with miRNAs (miRNA-Ref) and the other having a very low presence of these miRNAs. As shown in Fig. 6a, 130/plasma and 92/plasma arrays clustered together, showing a closer correspondence. This was confirmed by correlation analyses, showing better ρ values between 130/plasma and 92/plasma arrays (ρ = 0.961) (Fig. 6b) than those obtained between 130/plasma and 65/plasma arrays (ρ = 0.891) (Fig. 6c), or between 92/plasma and 65/plasma arrays (ρ = 0.928) (Fig. 6d). Taking 130/plasma as the gold standard, the sensitivity and specificity of the 92/plasma array were 0.928 and 0.995, respectively, whereas the same parameters for the 65/plasma array were 0.814 and 0.997, respectively. These results further confirm the overall better performance of 92 ng against 65 ng as starting amounts when conducting miRNA hybridization arrays. Specifically for plasma samples, the overall array performance when using 92 ng yielded acceptable results with high sensitivity and specificity, compared to the gold standard of 130 ng.

## Discussion

In recent years, microarray technology has become a common approach for identification of candidate biological markers. However, in the case of miRNAs and particularly of circulating miRNAs, an important shortcoming hampers widespread application of this technology: the extremely low concentration of these molecules in plasma may not yield the initial amount of miRNA content required by some hybridization array platforms. In this context, adequate miRNA quantification methods and confirmation of the potential for array platforms to use less starting material are warranted. This study sought to address both considerations.

Because differences in sample extraction, isolation, quantification, or profiling may yield controversial results^6, 19^, establishing standardized protocols is critical to obtain valid and reproducible results. Isolation and profiling of miRNAs from plasma can be extremely challenging, often due to the low concentration of these molecules in body fluids. We evaluated several commonly used quantification methods to assess the miRNA content in two dilutions of the miRNA-Ref and subsequently in plasma samples.

The results obtained from both types of samples suggest that spectrophotometer-based methodologies (Nanoquant and Nanodrop) overestimate miRNA quantification. In the case of miRNA-Ref samples, both methods procured miRNA-Ref values that were close to those expected for each working dilution but 3.5–6 times higher than those obtained by the Qubit method. In this context, there are two key considerations: 1) although miRNA-enriched, the miRNA-Ref contains total RNA from different human tissues or cell lines and therefore includes multiple RNA species, which implies that any truly miRNA-specific quantification method should show lower concentrations than those reported by the manufacturer for the RNA content of the miRNA-Ref dilutions; 2) the Nanoquant and Nanodrop assays base their quantification on absorbance; 260 nm-absorbance includes RNA, DNA, and free nucleotides, limiting the ability of these techniques to discriminate between these molecules. Therefore, within the values provided by both the Nanoquant and Nanodrop techniques, there is most likely a portion of molecules other than miRNAs (such as RNA and DNA) present in the miRNA-Ref. This hypothesis was confirmed by smear analyses of the Bio-PicoChip (0–4000 nt), with total RNA values close to those provided by the Nanodrop and Nanoquant, and the Bio-SmallChip (<200 nt), with RNA values closer to those obtained with the Qubit assay. In the case of plasma samples, the difference between Nanodrop and Nanoquant quantification values versus Qubit was even greater. Plasma samples are expected to carry additional contaminants from freeze-thaw cycles and the isolation process, such as other nucleotide molecules, phenols, or EDTA. Specifically, we used the miRNeasy kit from Qiagen, which requires Qiazol reagent, a phenol with 270 nm-absorbance and therefore susceptible to be quantified by the Nanoquant and Nanodrop assays when set for nucleotide detection at 260 nm absorbance^23, 26^. Accordingly, we could confirm the presence of abundant chemical and protein contaminants within the range of 230–280 nm absorbances in plasma samples. Unlike the Nanoquant and Nanodrop assays, the Qubit is not based on absorbance but instead uses specific fluorescent dyes selective for small RNA over other forms of RNA. One could speculate that using other RNA isolation methods more specific to miRNA would reduce the contaminant RNA species and could diminish the differences in quantification between the Qubit and spectrophotometer techniques (Nanoquant and Nanodrop). However, the miRNA extraction kit (miRNeasy, Qiagen) we selected has been recently shown to provide an optimal miRNA-enriched fraction from plasma^23^. On the other hand, using methods such as exosome extraction to enrich for small RNAs would not necessarily lead to a more accurate quantification by Nanoquant and Nanodrop, since current exosome isolation kits include all RNA species present in the vesicles (long RNAs such as mRNA and small RNAs such as non-coding RNA, miRNA, and others), which could potentially be detected by spectrophotometer readings. Despite its limitations, such as the potential to detect all small RNAs other than miRNAs, the fluorescent technology can minimize the effect of sample contamination by salt, proteins, degraded free-nucleotides, or long RNA or long DNA molecules^27^. Therefore, our results suggest that, particularly in plasma samples, the specific Qubit miRNA assay is likely to provide a more realistic estimation of miRNA quantification. On the other hand, our experience shows that Bioanalyzer technologies, particularly with low concentrations, provide highly variable quantification results and, although useful for analysis of RNA profile and integrity, do not seem a reliable method for miRNA quantification, particularly in plasma samples.

Another important aspect is that the Qubit assay for small RNAs offers a lower detection range compared to the other reliable platforms (0.05 ng/µL vs 3 ng/µL in the Nanoquant and 2 ng/µL in the Nanodrop), which is particularly important in material such as plasma samples with low miRNA content. This can explain why all three techniques correlate well as long as sample concentrations are higher than 2 ng/μL, but the correlation with Qubit is poor at concentrations below the lower detection threshold of the two absorbance-based platforms. Considering that concentration in our plasma samples ranged from 0.72 ng/µL to 1.65 ng/µL, the Qubit platform appears as the first-choice technique for plasma miRNA quantification. This platform has already been proposed for reliable quantification in other contexts: Mauger *et al*. used Qubit to quantify cell-free circulating DNA (Qubit dsDNA HS Assay)^28^, and Li *et al*. and Ge *et al*. proved its usefulness to measure all circulating RNA species (Qubit RNA HS Assay)^27, 29^, although none of these studies provided data on the equivalence or comparability to other quantification platforms. In the work by Deben *et al*., where different quantification platforms were compared, it was suggested that Qubit could be more suitable than spectrophotometer-based methods for RNA quantification in tissue samples^24^. However, to our knowledge no previous studies have attempted to comprehensively characterize the optimal methodology for miRNA quantification, and specifically in plasma samples.

Qubit detects all forms of small RNAs, and although miRNAs are the most abundant RNA species, other RNA forms were detected by RNA-seq. In our plasma samples, miRNAs accounted for 58.2% of all small RNAs, and in the miRNA-Ref, the portion of small RNAs corresponding to the miRNA region (20–40 nucleotides) was around 22%, with 84% of these being confirmed as miRNA molecules by RNA-seq of that specific region. These percentages can be of use to estimate the true miRNA content in a sample quantified with Qubit.

As noted, miRNA hybridization array platforms commonly require a specifically labelled minimal RNA content, which may be a limiting factor in the case of material with low miRNA concentration, such as plasma samples^22^. Due to the lack of validated quantification methods, and considering the limitations of plasma samples, many researchers fix a starting sample volume rather than a specific RNA amount^6^. This approach seems to be valid when using quantitative RT-PCR technology, which includes a pre-amplification step to overcome the limitations of low starting amounts, but is not generalizable to hybridization arrays. On the basis of these considerations, we tested the overall performance of the GeneChip platform, reducing the recommended starting amount of 130 ng of RNA to 92 ng (70%) and 65 ng (50%).

In three replicates of hybridization microarrays performed with 130 ng, 92 ng and 65 ng of miRNA-Ref (all quantified by Qubit), we observed expression of numerous miRNAs consistent with a sample enriched in miRNAs. Correlation between the three starting amounts was good (ρ = 0.994, 130/versus 92/miRNA-Ref arrays; ρ = 0.983, 130/versus 65/miRNA-Ref arrays). Taking 130/miRNA-Ref as the reference, the miRNA expression profile obtained was good (sensitivity 0.948 and specificity 0.996) with 65/miRNA-Ref, but better (0.978 and 0.997, respectively) for the 92/miRNA-Ref. In a second set of hybridization microarrays performed on plasma samples with the same three starting RNA amounts, only 118 miRNAs were found to be expressed, confirming the low expression of circulating miRNAs in plasma. In each case, the identical amounts of both types of samples loaded, together with the difference in the amounts of miRNAs expressed, can be explained by the presence of other contents in the plasma samples, such as small RNAs or even miRNAs poorly expressed (<3), which still would be detected by the Qubit assay at the time of quantification. In the case of plasma samples, correlation between 130/and 92/plasma arrays remained high (ρ = 0.961) but was lower between 130/and 65/plasma (ρ = 0.891). Accordingly, taking 130/plasma as the gold standard, sensitivity and specificity were 0.928 and 0.995 for the 92/plasma array, and 0.814 and 0.997 for the 65/plasma array, respectively.

Our results suggest that, even though specificity remains high when using the miRNA Affymetrix hybridization arrays with lower amounts of starting material (92 ng or 65 ng instead of the required 130 ng), sensitivity might be a limiting factor, particularly when working with the lowest concentration (65 ng) and in plasma samples. Therefore, it does not seem advisable to use the lowest amount in the study of circulating miRNA by hybridization arrays. Conversely, using 92 ng yielded acceptable sensitivity and specificity values compared to the gold-standard of 130 ng, even in plasma samples. This might be determinant because, whereas 92 ng (70%) of material can be easily obtained from 2-mL plasma samples, the usual volume obtained from conventional 4 mL-EDTA storage tubes, in our experience 130 ng (100%), is achieved in only 55% of cases.

In conclusion, the Qubit assay appeared to be the most appropriate method to estimate miRNA content in human plasma samples with very low miRNA concentration. Quantification by the Qubit platform allows more accurate estimation of the sample volume required to perform hybridization arrays. For arrays such as those from Affymetrix, it may be possible to use 70% of the theoretically required starting RNA amount when working with plasma samples, without significant loss of sensitivity and specificity. This finding may facilitate miRNA profiling by hybridization array technology when working with stored samples of limited volume and very low concentration of miRNAs such as the plasma samples collected for most clinical studies.

## Methods

### Blood samples

We collected 30 mL of peripheral blood from 12 healthy volunteers in EDTA tubes. Blood was rapidly centrifuged at 1500 g during 15 minutes at 4 °C to fractionate plasma from the buffy coat and the cellular fraction. Plasma from all donors was mixed together, generating a unique pool that was subsequently divided into independent aliquots (400–2000 µL each, tagged as 1Q to 20Q) and stored at −80 °C until needed. This strategy allowed us to study and compare identical plasma samples. All donors gave their written informed consent in accordance with the 2007 Spanish Law on Biomedical Research (14/2007), and the study was approved by the Clinical Research Ethical Committees of the Hospital del Mar Medical Research Institute (registration number 2013/5200/I).

### miRNA isolation

miRNA was isolated from each aliquot of plasma (1Q to 20Q) with the miRNeasy Serum/Plasma kit (Qiagen), following the manufacturer protocol. This kit was chosen because it is considered one of the best available methods to obtain an enriched miRNA fraction from plasma samples^23^. The kit combines a phenol/guanidine lysis of samples with silica-membrane columns to purify RNA, allowing the recovery of molecules smaller than 200 nucleotides from cell-free samples. This includes mostly miRNA but also other small RNA. The initial plasma aliquots were lysed and fractionated into 200 µL columns. RNA was eluted from each column by full-speed centrifugation for one minute in 14 µL of RNAse-free water. Final eluted volumes from each aliquot varied from 28 µL to 132 µL depending on the number of columns used.

### Universal Human miRNA Reference RNA

The Universal Human miRNA Reference from Agilent Technologies (miRNA-Ref) contains total RNA from nine human tissues or cell lines and is enriched with miRNAs. According to the manufacturer, this sample was obtained using a total RNA extraction kit, and so contains all forms of long and small RNA. Two working dilutions (10 ng/µL and 1 ng/µL) were used as internal controls for each quantification technique. These two concentrations cover the theoretical range of miRNA content in plasma^25^. The miRNA-Ref was also used for RNA-seq and to evaluate the performance of the microarray hybridization assays.

### RNA Integrity

The RNA profile and integrity of all samples (inferred by the RIN) was assessed using the Bioanalyzer 2100 (Agilent Technologies) with the Bio-PicoChip and Bio-SmallChip (see Quantification, Agilent 2100 Bioanalyzer).

### Quantification

#### Infinite^®^ 200 PRO Nanoquant

The Tecan Infinite^®^ 200 PRO Nanoquant platform is a full-spectrum absorbance-based spectrophotometer used to quantify DNA and RNA molecules, both absorbing at 260 nm, requiring a minimum nucleotide concentration of 3 ng/µL. The Nanoquant platform was used to quantify 3 µL of the two miRNA-Ref working dilutions and all plasma samples obtained from healthy controls (1Q to 20Q) according to manufacturer specifications.

#### Thermo Scientific^TM^ Nanodrop 2000

The Thermo Scientific^TM^ Nanodrop 2000 platform, also based on 260 nm-absorbance, and with a range of RNA detection from 2 ng/µL to 15 µg/µL, was used to quantify 1 µL of the two miRNA-Ref working dilutions and all plasma samples (1Q to 20Q).

#### Qubit^®^ Assay

The Qubit^®^ microRNA Assay kit, used with the Qubit^®^ 2.0 Fluorometer (Life Technologies) allows miRNA quantification by target-specific fluorescence detection, with a range from 0.05 ng/µL to 100 ng/µL, and thus is suitable for cell-free human plasma samples. The two miRNA-Ref working dilutions and all plasma samples (1Q to 20Q) were quantified following the manufacturer protocol (MAN0009427-Life Technologies): 1 µL or 10 µL (for 1 ng/µL miRNA-Ref) of the sample was diluted in 199 µL or 190 µL, respectively, of the working solution that contained the miRNA specific dye. The mix was vortexed and measured with the Qubit^®^ 2.0 Fluorometer’s RNA detection program after 2 min of incubation in the dark. Final concentration values expressed in ng/µL were obtained by applying the 1/200 dilution factor to the value given by the fluorometer. The 10 ng/µL miRNA-Ref dilution was used as internal control in every batch of measurements.

#### Agilent 2100 Bioanalyzer

Quantification was also assessed by performing a smear analysis of the electropherograms obtained by capillary electrophoresis with the Bio-PicoChip and the Bio-SmallChip. The Bio-PicoChip provides information on the total RNA profile, with a qualitative range from 50 pg/µL to 5 ng/µL, but no specific quantitative range. The Bio-SmallChip is specially indicated for small RNAs (length <200 nucleotides), with both quantitative and qualitative ranges from 50 pg/µL to 2 ng/µL. To perform these analyses, 1.1 µL of each sample was denatured during 2 minutes at 70 °C, followed by a 4 °C incubation for 2 minutes. To obtain the electrophoretic profile, 1 µL of each sample was loaded into the chip and processed following manufacturer specifications. When sample concentrations exceeded the upper detection threshold, these were diluted to fit the range and final sample concentration was calculated by applying the dilution factor to the value given by the Bioanalyzer.

### miRNA expression profiles

Expression miRNA profiles were checked using the hybridization GeneChip^®^ miRNA 3.0 and 4.0 high-density microarrays from Affymetrix. Specifically for the updated 4.0 version, the chip provides 100% coverage of the miRNAs in the miRBase database (v20), comprising probe sets for 2578 human mature miRNA, 2025 human pre-miRNA, and 1996 small cajal body-specific RNA (scaRNA) and small nucleolar RNA (snoRNA). According to the manufacturer protocol, the minimum amount of RNA needed to perform the hybridization process is 130 ng. We tested the microarray performance with 130 ng (100%), 92 ng (70%), and 65 ng (50%) of starting material using the miRNA-Ref samples, leading to the following study groups: 130/miRNA-Ref (n = 3), 92/miRNA-Ref (n = 3), 65/miRNA-Ref (n = 3). Furthermore, to validate our results in plasma samples and thus assess the clinical applicability of this strategy, we tested the microarray performance in plasma samples obtained from a unique pool of healthy controls (6Q–11Q mix) with the same three starting amounts: 130/plasma, 92/plasma, and 65/plasma. As Qubit provided the most accurate miRNA quantification, miRNA-Ref and plasma sample amounts were prepared according to the concentration provided by this platform. Following the manufacturer protocol, all samples were concentrated to a final working volume of 8 µL using a DNA SpeedVac System (DNA 120 Savant, ThermoFisher Scientific). Microarray miRNA expression profiles were obtained using the GeneChip^®^ miRNA 3.0 (for the miRNA-Ref samples) and 4.0 (for the 6Q–11Q mix of plasma samples) arrays. Briefly, all samples were poly(A)-tailed and biotin-ligated. Both reactions were performed using the Genisphere FlahTag™ Biotin HSR RNA Labelling Kit. After sample processing and before hybridization, biotin labelling was confirmed with the Enzyme Linked Oligosorbent Assay (ELOSA). All samples were then hybridized into the GeneChip^®^ miRNA 3.0 or 4.0 arrays during 16 hours at 48 °C and 60 rpm in a GeneChip^®^ Hybridization Oven 640. Following hybridization, the arrays were washed and stained in the GeneChip^®^ Fluidics Station 450, using the GeneChip^®^ Hybridization, Wash and Stain kit. The stained arrays were scanned with the GeneChip^®^ Scanner 3000 7 G, generating CEL files for each array. Data quality control was assessed using Affymetrix Expression Console software. All arrays met the quality control criteria.

### RNA sequencing

miRNA libraries were constructed following manufacturer specifications using the NEXTflex^TM^ Small RNA-seq Kit V3 (Bioo Scientific): 10 ng of RNA were subjected first to the 3’ 4 N Adapter ligation (1/4 dilution of the 4 N Adenylated Adapter), followed by the ligation of the 5’ 4 N Adapter, also diluted 1/4. Samples were then reverse transcribed and amplified by 25 PCR cycles, where barcode primers where added. Finally, a PAGE size selection was performed to recover fragments with more than 130 and less than 200 base pairs (containing all molecules between 20 and 40 nucleotides plus the adaptors) that were finally eluted in 12 µL of the resuspension buffer. These were quantified using qPCR with the Takara Library Quantification Kit (Takara Clontech) and sequenced in an Illumina Miseq system.

### Small RNA-seq data processing

Reads were initially trimmed for TGGAATTCTCGGGTGCCAAGG adapters with cutadapt. Resulting inserts smaller than 15 nt were excluded. Four bases were then trimmed from each side of the read, with these parameters: -u 4 -u -4. Trimmed reads were mapped to the human genome (assembly hg38) using STAR v2.5.1b, with these parameters:–outFilterMultimapNmax 2–outFilterMultimapScoreRange 0–outFilterScoreMinOverLread 0–outFilterMatchNminOverLread 0–outFilterMatchNmin 16–outFilterMismatchNmax 1–alignSJDBoverhangMin 1000–alignIntronMax 1. Bedtools was used to count the number of reads mapping on each annotated feature from Gencode annotation, version 25 without long RNAs.

### Statistical analysis

Quantification results are presented as mean, standard deviation (SD), and CV. Correlations between different quantification platforms were assessed using the Spearman rank correlation coefficient (ρ). Regression lines were also generated with their 95% confidence interval and 95% prediction interval.

Data from the miRNA expression profiles for the miRNA-Ref and plasma were quality-checked in the Expression Console and normalized independently using the human probes and the Robust Multichip Average (RMA) methodology in the Expression Console. Once normalized, data were imported in R (v3.1.1), where all analyses were performed. MiRNA-Ref data were corrected for batch effects using the ComBat method from sva package. Heat maps were constructed using the gplots package. A miRNA was considered to be expressed when its log2 expression was greater than 3. In particular, we defined the gold standard to be miRNAs expressed by means of the 130 ng starting amount microarray data (mean of the three 130 replicates of miRNA-Ref and 130/plasma).

## Electronic supplementary material

Supplementary Information

## References

[1] Bartel DP (2009). MicroRNAs: target recognition and regulatory functions. Cell.

[2] Fabian MR, Sonenberg N, Filipowicz W (2010). Regulation of mRNA translation and stability by microRNAs. Annu. Rev. Biochem..

[3] Lewis BP, Burge CB, Bartel DP (2005). Conserved seed pairing, often flanked by adenosines, indicates that thousands of human genes are microRNA targets. Cell.

[4] Blondal T (2013). Assessing sample and miRNA profile quality in serum and plasma or other biofluids. Methods.

[5] Li Y, Kowdley KV (2012). Method for microRNA isolation from clinical serum samples. Anal. Biochem..

[6] Moldovan L (2014). Methodological challenges in utilizing miRNAs as circulating biomarkers. J. Cell. Mol. Med..

[7] Kroh EM, Parkin RK, Mitchell PS, Tewari M (2010). Analysis of circulating microRNA biomarkers in plasma and serum using quantitative reverse transcription-PCR (qRT-PCR). Methods.

[8] Cortez MA (2011). MicroRNAs in body fluids–the mix of hormones and biomarkers. Nat. Rev. Clin. Oncol..

[9] Burgos KL (2013). Identification of extracellular miRNA in human cerebrospinal fluid by next-generation sequencing. RNA.

[10] Creemers EE, Tijsen AJ, Pinto YM (2012). Circulating microRNAs: novel biomarkers and extracellular communicators in cardiovascular disease?. Circ. Res..

[11] Mitchell PS (2008). Circulating microRNAs as stable blood-based markers for cancer detection. Proc. Natl. Acad. Sci. U. S. A..

[12] Duttagupta R, Jiang R, Gollub J, Getts RC, Jones KW (2011). Impact of cellular miRNAs on circulating miRNA biomarker signatures. PLoS One.

[13] Jung M (2010). Robust microRNA stability in degraded RNA preparations from human tissue and cell samples. Clin. Chem..

[14] Jensen SG (2011). Evaluation of two commercial global miRNA expression profiling platforms for detection of less abundant miRNAs. BMC Genomics.

[15] Mestdagh P (2014). Evaluation of quantitative miRNA expression platforms in the microRNA quality control (miRQC) study. Nat. Methods.

[16] Cheng G (2015). Circulating miRNAs: Roles in cancer diagnosis, prognosis and therapy. Adv. Drug Deliv. Rev..

[17] Wang, F., Chen, C. & Wang, D. Circulating microRNAs in cardiovascular diseases: from biomarkers to therapeutic targets. *Front*. *Med*., doi:10.1007/s11684-014-0379-2 (2014). 10.1007/s11684-014-0379-2 25445171

[18] Cheng HH (2013). Plasma processing conditions substantially influence circulating microRNA biomarker levels. PLoS One.

[19] Witwer, K. W. C MRNA Biomarker Studies: Pitfalls and Potential Solutions. *Clin*. *Chem*., doi:10.1373/clinchem.2014.221341 (2014). 10.1373/clinchem.2014.221341 25391989

[20] Monleau M (2014). Comparison of different extraction techniques to profile microRNAs from human sera and peripheral blood mononuclear cells. BMC Genomics.

[21] Wang K (2012). Comparing the MicroRNA spectrum between serum and plasma. PLoS One.

[22] Moret I (2013). Assessing an improved protocol for plasma microRNA extraction. PLoS One.

[23] El-Khoury V, Pierson S, Kaoma T, Bernardin F, Berchem G (2016). Assessing cellular and circulating miRNA recovery: the impact of the RNA isolation method and the quantity of input material. Sci. Rep..

[24] Deben C (2013). Expression analysis on archival material revisited: isolation and quantification of RNA extracted from FFPE samples. Diagn. Mol. Pathol..

[25] Pritchard CC, Cheng HH, Tewari M (2012). MicroRNA profiling: approaches and considerations. Nat. Rev. Genet..

[26] Lee JTY, Cheung KMC, Leung VYL (2014). Correction for concentration overestimation of nucleic acids with phenol. Anal. Biochem..

[27] Li X, Bn-Dov IZ, Mauro M, Williams Z (2015). Lwering the quantification limit of the QubitTM RNA HS Assay using RNA spike-in. BMC Mol. Biol..

[28] Mauger, F., Dulary, C., Daviaud, C., Deleuze, J.-F. & Tost, J. Comprehensive evaluation of methods to isolate, quantify, and characterize circulating cell-free DNA from small volumes of plasma. *Anal*. *Bioanal*. *Chem*. 6873–6878, doi:10.1007/s00216-015-8846-4 (2015). 10.1007/s00216-015-8846-4 26123439

[29] Ge Q (2014). MiRNA in plasma exosome is stable under different storage conditions. Molecules.

