# Supplementary material for: Defining quantification methods and optimizing protocols for microarray hybridization of circulating microRNAs
Source: Sci Rep. 2017 Aug 10;7:7725. doi: 10.1038/s41598-017-08134-3 (PMC5552704; doi:10.1038/s41598-017-08134-3)
Supplement: Supplementary file 1 — Supplementary Information [file 41598_2017_8134_MOESM1_ESM.pdf]

SUPPLEMENTARY INFORMATION

Defining quantification methods and optimizing protocols for microarray hybridization of circulating microRNAs.

Anna Garcia-Elias, Leonor Alloza, Eulàlia Puigdecanet, Lara Nonell, Marta Tajes, Joao Curado, Cristina Enjuanes, Oscar Díaz, Jordi Bruguera, Julio Martí-Almor, Josep Comín-Colet, Begoña Benito.

Table S1. RNA contents in five different concentrations of miRNA-Ref samples.

Table S2. 260/230 and 260/280 ratios provided by the Nanodrop on miRNA-Ref and plasma samples at different concentrations.

Table S3. RNA contents in five different concentrations of plasma samples.

Table S4. Percentage of miRNA in miRNA-Ref samples according to the Bio-SmallChip electropherograms.

Table S5. List of miRNAs detected in the miRNA-Ref arrays.

Table S6. List of miRNAs detected in the human plasma arrays.

Table S1: RNA contents in five different concentrations of miRNA-Ref

	Quantification platforms (ng/µL)							
	**Nanoquant**	*normalized (*)*	**Nanodrop**	*normalized (*)*	**Qubit**	*normalized (*)*	**Bio-PicoChip**	*normalized (*)*
	9.52	1.00	9.40	1.00	2.71	1.00	8.37	1.00
	9.12	0.96	9.60	1.02	2.69	0.99	8.32	1.02
	9.60	1.01	8.60	0.91	2.61	0.96	8.03	0.97
Mean (SD)	***9.41 (0.26)***		***9.20 (0.53)***		***2.67 (0.05)***		***8.24 (0.18)***	
CV (%)	***2.73***		***5.75***		***1.98***		***2.20***	
	22.40	2.35	22.60	2.40	6.64	2.45	20.12	2.44
	22.56	2.37	22.40	2.38	6.79	2.51	18.66	2.26
	22.88	2.40	22.30	2.37	6.43	2.37	20.09	2.44
Mean (SD)	***22.61 (0.24)***		***22.43 (0.15)***		***6.62 (0.18)***		***19.62 (0.84)***	
CV (%)	***1.08***		***0.68***		***2.73***		***4.26***	
	29.92	3.14	30.20	3.21	8.93	3.30	33.72	4.09
	30.96	3.25	29.90	3.18	8.64	3.19	32.63	3.96
	29.52	3.10	29.70	3.16	8.28	3.06	33.69	4.09
Mean (SD)	***30.13 (0.74)***		***29.93 (0.21)***		***8.62 (0.33)***		***33.35 (0.62)***	
CV (%)	***2.47***		***0.69***		***3.78***		***1.86***	
	39.76	4.18	40.20	4.28	12.59	4.65	43.19	5.24
	39.60	4.16	40.30	4.29	12.24	4.52	41.80	5.07
	39.04	4.10	40.00	4.26	12.59	4.65	43.00	5.22
Mean (SD)	***39.47 (0.38)***		***40.17 (0.15)***		***12.47 (0.20)***		***42.66 (0.76)***	
CV (%)	***1.00***		***0.40***		***1.60***		***1.77***	
	46.00	4.83	46.60	4.96	15.61	5.76	46.97	5.70
	46.00	4.83	46.60	4.96	15.05	5.55	48.60	5.90
	45.28	4.76	46.90	4.99	15.52	5.73	46.80	5.68
Mean (SD)	***45.76 (0.42)***		***46.70 (0.17)***		***15.39 (0.30)***		***47.46 (0.99)***	
CV (%)	***0.91***		***0.37***		***1.95***		***2.10***	

Table S2: 260/230 and 260/280 ratios of miRNA-Ref and plasma samples

	miRNA-Ref			plasma samples		
	Nanodrop (ng/μL)	***260/230 ratio***	***260/280 ratio***	Nanodrop (ng/μL)	***260/230 ratio***	***260/280 ratio***
	9.40	2.03	1.93	18.40	0.17	1.28
	9.60	2.29	1.78	16.20	0.16	1.27
	8.60	2.25	2.31	19.80	0.19	1.26
	22.60	1.94	2.03	27.80	0.14	1.21
	22.40	2.03	1.96	26.60	0.14	1.20
	22.30	1.91	1.86	27.60	0.14	1.21
	30.20	1.93	1.95	39.80	0.13	1.22
	29.90	1.94	2.01	36.50	0.12	1.22
	29.70	1.95	2.02	41.20	0.13	1.22
	40.20	1.99	1.97	54.50	0.15	1.21
	40.30	1.91	1.99	50.30	0.14	1.21
	40.00	1.89	1.97	46.40	0.13	1.21
	46.60	1.95	1.99	61.10	0.14	1.19
	46.60	1.94	1.98	57.40	0.13	1.19
	46.90	1.94	1.99	62.60	0.14	1.17
Mean (SD)		***1.99 (0.12)***	***1.98 (0.11)***		***0.14 (0.02)***	***1.22 (0.03)***

Table S3: RNA contents in five different concentrations of plasma samples

	Quantification platforms (ng/µL)							
	**Nanoquant**	*normalized (*)*	**Nanodrop**	*normalized (*)*	**Qubit**	*normalized (*)*	**Bio-PicoChip**	*normalized (*)*
	13.92	1.05	18.40	1.00	3.44	1.48	0.330	1.00
	13.28	1.00	16.20	0.88	1.77	0.76	0.228	0.69
	13.92	1.05	19.80	1.08	2.33	1.00	0.249	0.75
Mean (SD)	***13.71 (0.37)***		***18.13 (1.81)***		***2.51 (0.85)***		***0.269 (0.054)***	
CV (%)	***2.70***		***10.01***		***33.82***		***20.02***	
	19.52	1.47	27.80	1.51	4.16	1.79	0.330	1.00
	19.04	1.43	26.60	1.45	4.07	1.75	---	---
	18.88	1.42	27.60	1.50	3.95	1.70	0.275	0.83
Mean (SD)	***19.15 (0.33)***		***27.33 (0.64)***		***4.06 (0.10)***		***0.303 (0.039)***	
CV (%)	***1.74***		***2.35***		***2.59***		***12.86***	
	28.64	2.16	39.80	2.16	5.88	2.52	0.312	0.95
	28.40	2.14	36.50	1.98	5.77	2.48	0.351	1.06
	27.60	2.08	41.20	2.24	5.68	2.44	0.366	1.11
Mean (SD)	***28.21 (0.54)***		***39.17 (2.41)***		***5.78 (0.10)***		***0.343 (0.028)***	
CV (%)	***1.93***		***6.16***		***1.73***		***8.13***	
	35.28	2.66	54.50	2.96	7.19	3.09	0.320	0.97
	36.88	2.78	50.30	2.73	7.33	3.15	0.340	1.03
	32.64	2.46	46.40	2.52	7.44	3.19	---	---
Mean (SD)	***34.93 (2.14)***		***50.40 (4.05)***		***7.32 (0.13)***		***0.330 (0.014)***	
CV (%)	***6.13***		***8.04***		***1.71***		***4.29***	
	39.60	2.98	61.10	3.32	9.11	3.91	0.403	1.22
	39.12	2.95	57.40	3.12	9.22	3.96	0.222	0.67
	34.24	2.58	62.60	3.40	9.08	3.90	0.385	1.17
Mean (SD)	***37.65 (2.97)***		***60.37 (2.68)***		***9.14 (0.07)***		***0.337 (0.100)***	
CV (%)	***7.88***		***4.43***		***0.81***		***29.62***	

Table 4: Percentage of miRNA in miRNA-Ref samples

10 ng/µL working dilution	% of miRNA
1	21
2	21
3	20
4	21
5	23
6	23
7	25
8	24
9	23
10	23
Mean (SD)	22 (1.58)

Table S5: List of miRNAs detected in the miRNA-Ref arrays

hsa-mir-1224	hsa-mir-523	hsa-miR-1228
hsa-mir-124-1	hsa-mir-525	hsa-miR-122
hsa-mir-124-2	hsa-mir-525	hsa-miR-1231
hsa-mir-124-3	hsa-mir-548ai	hsa-miR-1238
hsa-mir-197	hsa-mir-548ai	hsa-miR-124-star
hsa-mir-200b	hsa-mir-550a-1	hsa-miR-1246
hsa-mir-20b	hsa-mir-550a-2	hsa-miR-1247
hsa-mir-20b	hsa-mir-550a-3	hsa-miR-124
hsa-mir-214	hsa-mir-572	hsa-miR-125a-3p
hsa-mir-23a	hsa-mir-629	hsa-miR-125a-5p
hsa-mir-302c	hsa-mir-663	hsa-miR-125b-1-star
hsa-mir-30c-2	hsa-mir-885	hsa-miR-125b-2-star
hsa-mir-3154	hsa-mir-933	hsa-miR-125b
hsa-mir-3180-1	hsa-mir-935	hsa-miR-1260b
hsa-mir-3180-3	hsa-mir-941-3	hsa-miR-1268
hsa-mir-3180-4	hsa-mir-941-4	hsa-miR-1268b
hsa-mir-339	hsa-mir-99b	hsa-miR-126
hsa-mir-3648	hsa-let-7a	hsa-miR-127-3p
hsa-mir-3656	hsa-let-7b	hsa-miR-1271
hsa-mir-3676	hsa-let-7c	hsa-miR-1275
hsa-mir-3679	hsa-let-7d-star	hsa-miR-1280
hsa-mir-3687	hsa-let-7d	hsa-miR-1281
hsa-mir-3960	hsa-let-7e	hsa-miR-1287
hsa-mir-423	hsa-let-7f	hsa-miR-128
hsa-mir-4449	hsa-let-7g	hsa-miR-129-3p
hsa-mir-4466	hsa-let-7i	hsa-miR-129-5p
hsa-mir-4469	hsa-miR-100	hsa-miR-1296
hsa-mir-4485	hsa-miR-103a	hsa-miR-1301
hsa-mir-4523	hsa-miR-105	hsa-miR-1307
hsa-mir-4634	hsa-miR-106a	hsa-miR-130a
hsa-mir-4758	hsa-miR-106b-star	hsa-miR-130b
hsa-mir-4758	hsa-miR-106b	hsa-miR-1323
hsa-mir-4785	hsa-miR-107	hsa-miR-132
hsa-mir-4787	hsa-miR-10a	hsa-miR-133a
hsa-mir-485	hsa-miR-10b-star	hsa-miR-133b
hsa-mir-500b	hsa-miR-10b	hsa-miR-134
hsa-mir-502	hsa-miR-1180	hsa-miR-135a-star
hsa-mir-5095	hsa-miR-1202	hsa-miR-137
hsa-mir-516a-1	hsa-miR-1207-5p	hsa-miR-138-1-star
hsa-mir-516a-2	hsa-miR-1224-5p	hsa-miR-138-2-star
hsa-mir-516b-1	hsa-miR-1225-5p	hsa-miR-138
hsa-mir-518e	hsa-miR-1226	hsa-miR-139-3p
hsa-mir-520g	hsa-miR-1228-star	hsa-miR-139-5p

hsa-miR-140-3p
hsa-miR-140-5p
hsa-miR-141
hsa-miR-143-star
hsa-miR-143
hsa-miR-145
hsa-miR-1469
hsa-miR-146a
hsa-miR-146b-5p
hsa-miR-148a
hsa-miR-148b
hsa-miR-149-star
hsa-miR-149
hsa-miR-150-star
hsa-miR-150
hsa-miR-151-3p
hsa-miR-151-5p
hsa-miR-151b
hsa-miR-152
hsa-miR-153
hsa-miR-154
hsa-miR-155
hsa-miR-1587
hsa-miR-15a
hsa-miR-15b
hsa-miR-16
hsa-miR-17-star
hsa-miR-17
hsa-miR-181a-2-star
hsa-miR-181a-star
hsa-miR-181a
hsa-miR-181b
hsa-miR-181c-star
hsa-miR-181c
hsa-miR-181d
hsa-miR-1825
hsa-miR-182
hsa-miR-183
hsa-miR-184
hsa-miR-185
hsa-miR-187
hsa-miR-188-5p
hsa-miR-18a-star
hsa-miR-18a
hsa-miR-18b
hsa-miR-1908
hsa-miR-1909
hsa-miR-191-star
hsa-miR-1910
hsa-miR-1915
hsa-miR-191
hsa-miR-192-star
hsa-miR-192
hsa-miR-193a-5p
hsa-miR-193b-star
hsa-miR-193b
hsa-miR-194-star
hsa-miR-194
hsa-miR-195
hsa-miR-196a
hsa-miR-196b
hsa-miR-1972
hsa-miR-1973
hsa-miR-197
hsa-miR-199a-3p
hsa-miR-199a-5p
hsa-miR-199b-3p
hsa-miR-199b-5p
hsa-miR-19a
hsa-miR-19b
hsa-miR-1
hsa-miR-200a-star
hsa-miR-200a
hsa-miR-200b-star
hsa-miR-200b
hsa-miR-200c
hsa-miR-202
hsa-miR-203
hsa-miR-204
hsa-miR-205
hsa-miR-206
hsa-miR-20a
hsa-miR-20b-star
hsa-miR-20b
hsa-miR-210
hsa-miR-2110
hsa-miR-212
hsa-miR-214-star
hsa-miR-214
hsa-miR-215
hsa-miR-216b
hsa-miR-217
hsa-miR-219-2-3p
hsa-miR-21
hsa-miR-22-star
hsa-miR-221
hsa-miR-222
hsa-miR-223
hsa-miR-224-star
hsa-miR-224
hsa-miR-2277-5p
hsa-miR-22
hsa-miR-2392
hsa-miR-23a-star
hsa-miR-23a
hsa-miR-23b-star
hsa-miR-23b
hsa-miR-23c
hsa-miR-24-2-star
hsa-miR-24
hsa-miR-25-star
hsa-miR-25
hsa-miR-26a
hsa-miR-26b
hsa-miR-27a-star
hsa-miR-27a
hsa-miR-27b-star
hsa-miR-27b
hsa-miR-28-3p
hsa-miR-28-5p
hsa-miR-2861
hsa-miR-296-3p
hsa-miR-299-3p
hsa-miR-299-5p
hsa-miR-29a
hsa-miR-29b-1-star
hsa-miR-29b-2-star
hsa-miR-29b
hsa-miR-29c-star
hsa-miR-29c
hsa-miR-302a-star
hsa-miR-302a
hsa-miR-302b
hsa-miR-302c-star
hsa-miR-302c
hsa-miR-302d-star
hsa-miR-302d
hsa-miR-30a-star

hsa-miR-30a
hsa-miR-30b-star
hsa-miR-30b
hsa-miR-30c-2-star
hsa-miR-30c
hsa-miR-30d
hsa-miR-30e-star
hsa-miR-30e
hsa-miR-31-star
hsa-miR-3124-5p
hsa-miR-3135b
hsa-miR-3141
hsa-miR-3162-5p
hsa-miR-3175
hsa-miR-3178
hsa-miR-3180-3p
hsa-miR-3180
hsa-miR-3185
hsa-miR-3188
hsa-miR-3195
hsa-miR-3196
hsa-miR-3197
hsa-miR-31
hsa-miR-3200-3p
hsa-miR-320a
hsa-miR-320b
hsa-miR-320c
hsa-miR-320d
hsa-miR-320e
hsa-miR-323-3p
hsa-miR-324-3p
hsa-miR-324-5p
hsa-miR-328
hsa-miR-329
hsa-miR-330-3p
hsa-miR-331-3p
hsa-miR-331-5p
hsa-miR-335
hsa-miR-337-5p
hsa-miR-338-5p
hsa-miR-339-3p
hsa-miR-339-5p
hsa-miR-342-3p
hsa-miR-342-5p
hsa-miR-345
hsa-miR-346
hsa-miR-34a
hsa-miR-34b-star
hsa-miR-34c-3p
hsa-miR-34c-5p
hsa-miR-3607-5p
hsa-miR-3609
hsa-miR-361-5p
hsa-miR-3613-3p
hsa-miR-3615
hsa-miR-3619-5p
hsa-miR-362-5p
hsa-miR-3621
hsa-miR-363
hsa-miR-3648
hsa-miR-3651
hsa-miR-3652
hsa-miR-3656
hsa-miR-3663-3p
hsa-miR-3665
hsa-miR-3679-5p
hsa-miR-3687
hsa-miR-370
hsa-miR-371-3p
hsa-miR-371-5p
hsa-miR-371b-5p
hsa-miR-372
hsa-miR-373
hsa-miR-374b
hsa-miR-375
hsa-miR-376a
hsa-miR-376c
hsa-miR-377-star
hsa-miR-378-star
hsa-miR-378
hsa-miR-378b
hsa-miR-378c
hsa-miR-378d
hsa-miR-378e
hsa-miR-378f
hsa-miR-378g
hsa-miR-378h
hsa-miR-378i
hsa-miR-379
hsa-miR-381
hsa-miR-382
hsa-miR-383
hsa-miR-3911
hsa-miR-3937
hsa-miR-3940-5p
hsa-miR-3960
hsa-miR-409-3p
hsa-miR-409-5p
hsa-miR-410
hsa-miR-411-star
hsa-miR-411
hsa-miR-421
hsa-miR-422a
hsa-miR-423-3p
hsa-miR-423-5p
hsa-miR-424-star
hsa-miR-424
hsa-miR-425-star
hsa-miR-4253
hsa-miR-425
hsa-miR-4269
hsa-miR-4270
hsa-miR-4281
hsa-miR-4284
hsa-miR-4286
hsa-miR-4298
hsa-miR-4306
hsa-miR-4310
hsa-miR-4317
hsa-miR-431
hsa-miR-4322
hsa-miR-4324
hsa-miR-4329
hsa-miR-432
hsa-miR-433
hsa-miR-4417
hsa-miR-4429
hsa-miR-4430
hsa-miR-4433
hsa-miR-4443
hsa-miR-4449
hsa-miR-4454
hsa-miR-4458
hsa-miR-4459
hsa-miR-4462
hsa-miR-4463
hsa-miR-4465
hsa-miR-4466

hsa-miR-4467
hsa-miR-4484
hsa-miR-4485
hsa-miR-4486
hsa-miR-4487
hsa-miR-4488
hsa-miR-4492
hsa-miR-4497
hsa-miR-4498
hsa-miR-449a
hsa-miR-449b-star
hsa-miR-449b
hsa-miR-449c
hsa-miR-4505
hsa-miR-4507
hsa-miR-4508
hsa-miR-4510
hsa-miR-4516
hsa-miR-451
hsa-miR-4521
hsa-miR-452
hsa-miR-4530
hsa-miR-4532
hsa-miR-4534
hsa-miR-4539
hsa-miR-454
hsa-miR-455-3p
hsa-miR-4634
hsa-miR-4640-5p
hsa-miR-4649-5p
hsa-miR-4651
hsa-miR-4655-5p
hsa-miR-4656
hsa-miR-4665-5p
hsa-miR-4668-5p
hsa-miR-4669
hsa-miR-4674
hsa-miR-4685-5p
hsa-miR-4687-3p
hsa-miR-4688
hsa-miR-4689
hsa-miR-4690-5p
hsa-miR-4695-5p
hsa-miR-4706
hsa-miR-4707-5p
hsa-miR-4710
hsa-miR-4721
hsa-miR-4728-5p
hsa-miR-4732-5p
hsa-miR-4734
hsa-miR-4739
hsa-miR-4741
hsa-miR-4743
hsa-miR-4745-5p
hsa-miR-4749-5p
hsa-miR-4750
hsa-miR-4758-5p
hsa-miR-4763-3p
hsa-miR-4764-5p
hsa-miR-4787-5p
hsa-miR-4800-3p
hsa-miR-483-5p
hsa-miR-484
hsa-miR-485-3p
hsa-miR-485-5p
hsa-miR-486-3p
hsa-miR-486-5p
hsa-miR-487a
hsa-miR-487b
hsa-miR-489
hsa-miR-490-5p
hsa-miR-491-5p
hsa-miR-493-star
hsa-miR-493
hsa-miR-494
hsa-miR-495
hsa-miR-497
hsa-miR-498
hsa-miR-499-5p
hsa-miR-500a-star
hsa-miR-500a
hsa-miR-501-3p
hsa-miR-501-5p
hsa-miR-502-3p
hsa-miR-503
hsa-miR-504
hsa-miR-505-star
hsa-miR-505
hsa-miR-506
hsa-miR-508-3p
hsa-miR-508-5p
hsa-miR-509-3-5p
hsa-miR-509-3p
hsa-miR-509-5p
hsa-miR-510
hsa-miR-512-3p
hsa-miR-512-5p
hsa-miR-513a-5p
hsa-miR-513b
hsa-miR-513c
hsa-miR-514
hsa-miR-514b-5p
hsa-miR-515-3p
hsa-miR-515-5p
hsa-miR-516a-5p
hsa-miR-516b
hsa-miR-517-star
hsa-miR-517a
hsa-miR-517b
hsa-miR-517c
hsa-miR-518a-3p
hsa-miR-518a-5p
hsa-miR-518b
hsa-miR-518c-star
hsa-miR-518d-5p
hsa-miR-518e-star
hsa-miR-518e
hsa-miR-518f-star
hsa-miR-518f
hsa-miR-519a-star
hsa-miR-519a
hsa-miR-519b-3p
hsa-miR-519b-5p
hsa-miR-519c-3p
hsa-miR-519c-5p
hsa-miR-519d
hsa-miR-519e-star
hsa-miR-520a-3p
hsa-miR-520a-5p
hsa-miR-520c-5p
hsa-miR-520d-3p
hsa-miR-520d-5p
hsa-miR-520g
hsa-miR-520h
hsa-miR-522-star
hsa-miR-522
hsa-miR-523-star
hsa-miR-523

hsa-miR-524-3p
hsa-miR-524-5p
hsa-miR-525-3p
hsa-miR-525-5p
hsa-miR-526a
hsa-miR-526b
hsa-miR-527
hsa-miR-532-3p
hsa-miR-532-5p
hsa-miR-542-5p
hsa-miR-543
hsa-miR-548ak
hsa-miR-550a-star
hsa-miR-550a
hsa-miR-551b-star
hsa-miR-572
hsa-miR-574-3p
hsa-miR-574-5p
hsa-miR-578
hsa-miR-584
hsa-miR-598
hsa-miR-602
hsa-miR-625
hsa-miR-628-3p
hsa-miR-629-star
hsa-miR-629
hsa-miR-638
hsa-miR-642b
hsa-miR-652
hsa-miR-654-3p
hsa-miR-654-5p
hsa-miR-660
hsa-miR-663
hsa-miR-664-star
hsa-miR-665
hsa-miR-671-5p
hsa-miR-675-star
hsa-miR-675
hsa-miR-7-1-star
hsa-miR-708
hsa-miR-720
hsa-miR-744
hsa-miR-760
hsa-miR-762
hsa-miR-766
hsa-miR-767-5p
hsa-miR-769-5p
hsa-miR-770-5p
hsa-miR-874
hsa-miR-875-3p
hsa-miR-877
hsa-miR-885-3p
hsa-miR-885-5p
hsa-miR-891a
hsa-miR-9-star
hsa-miR-92a-1-star
hsa-miR-92a
hsa-miR-92b-star
hsa-miR-92b
hsa-miR-93-star
hsa-miR-933
hsa-miR-934
hsa-miR-935
hsa-miR-939
hsa-miR-93
hsa-miR-940
hsa-miR-941
hsa-miR-98
hsa-miR-99a
hsa-miR-99b-star
hsa-miR-99b
hsa-miR-9

Table S6: List of miRNAs detected in the human plasma arrays

hsa-let-7b-5p	hsa-miR-4484	hsa-miR-663a
hsa-miR-1227-5p	hsa-miR-4487	hsa-mir-6722
hsa-miR-1228-5p	hsa-miR-4488	hsa-miR-6722-3p
hsa-miR-1237-5p	hsa-miR-4492	hsa-miR-6724-5p
hsa-miR-1268a	hsa-miR-4497	hsa-miR-6727-5p
hsa-miR-1268b	hsa-miR-4508	hsa-miR-6729-5p
hsa-miR-1273g-3p	hsa-miR-4516	hsa-miR-6732-5p
hsa-miR-1281	hsa-miR-4529-3p	hsa-miR-6750-5p
hsa-miR-1343-5p	hsa-miR-4530	hsa-miR-6752-5p
hsa-miR-1469	hsa-miR-4532	hsa-miR-6756-5p
hsa-miR-149-3p	hsa-miR-4632-5p	hsa-miR-6765-5p
hsa-miR-1908-5p	hsa-miR-4668-5p	hsa-miR-6771-5p
hsa-miR-1915-3p	hsa-miR-4687-3p	hsa-miR-6775-5p
hsa-miR-2277-5p	hsa-miR-4695-5p	hsa-mir-6776
hsa-miR-2861	hsa-miR-4701-3p	hsa-miR-6777-5p
hsa-miR-297	hsa-miR-4706	hsa-miR-6779-5p
hsa-miR-3064-5p	hsa-miR-4707-5p	hsa-miR-6786-5p
hsa-miR-3128	hsa-miR-4739	hsa-miR-6789-5p
hsa-miR-3141	hsa-miR-4745-5p	hsa-miR-6791-5p
hsa-miR-3148	hsa-miR-4763-3p	hsa-miR-6794-5p
hsa-miR-3196	hsa-miR-4787-5p	hsa-mir-6800
hsa-miR-3201	hsa-miR-486-5p	hsa-miR-6800-5p
hsa-miR-320a	hsa-miR-5001-5p	hsa-miR-6803-5p
hsa-miR-320b	hsa-mir-520g	hsa-miR-6805-5p
hsa-miR-320c	hsa-mir-520h	hsa-miR-6816-5p
hsa-miR-320d	hsa-mir-550a-1	hsa-miR-6821-5p
hsa-miR-320e	hsa-mir-550a-2	hsa-miR-6850-5p
hsa-miR-3613-3p	hsa-mir-550a-3	hsa-miR-6858-5p
hsa-miR-3620-5p	hsa-miR-5787	hsa-miR-6869-5p
hsa-miR-3621	hsa-miR-595	hsa-miR-7108-5p
hsa-miR-3656	hsa-miR-6068	hsa-miR-7110-5p
hsa-miR-3665	hsa-miR-6085	hsa-mir-7515
hsa-miR-378h	hsa-miR-6087	hsa-miR-762
hsa-miR-3940-5p	hsa-miR-6088	hsa-miR-7704
hsa-miR-3960	hsa-miR-6089	hsa-miR-8069
hsa-miR-4281	hsa-miR-6090	hsa-miR-8072
hsa-miR-4429	hsa-miR-6125	hsa-miR-8075
hsa-miR-4440	hsa-miR-6126	hsa-miR-8084
hsa-miR-4466	hsa-miR-638	
hsa-miR-4467	hsa-miR-6511b-5p	
